# A New Strategy for Analyzing Time-Series Data Using Dynamic Networks: Identifying Prospective Biomarkers of Hepatocellular Carcinoma

**DOI:** 10.1038/srep32448

**Published:** 2016-08-31

**Authors:** Xin Huang, Jun Zeng, Lina Zhou, Chunxiu Hu, Peiyuan Yin, Xiaohui Lin

**Affiliations:** 1 School of Computer Science & Technology, Dalian University of Technology, 116024 Dalian, China; 2 Key Laboratory of Separation Science for Analytical Chemistry, Dalian Institute of Chemical Physics, Chinese Academy of Sciences, Dalian 116023, China

## Abstract

Time-series metabolomics studies can provide insight into the dynamics of disease development and facilitate the discovery of prospective biomarkers. To improve the performance of early risk identification, a new strategy for analyzing time-series data based on dynamic networks (ATSD-DN) in a systematic time dimension is proposed. In ATSD-DN, the non-overlapping ratio was applied to measure the changes in feature ratios during the process of disease development and to construct dynamic networks. Dynamic concentration analysis and network topological structure analysis were performed to extract early warning information. This strategy was applied to the study of time-series lipidomics data from a stepwise hepatocarcinogenesis rat model. A ratio of lyso-phosphatidylcholine (LPC) 18:1/free fatty acid (FFA) 20:5 was identified as the potential biomarker for hepatocellular carcinoma (HCC). It can be used to classify HCC and non-HCC rats, and the area under the curve values in the discovery and external validation sets were 0.980 and 0.972, respectively. This strategy was also compared with a weighted relative difference accumulation algorithm (wRDA), multivariate empirical Bayes statistics (MEBA) and support vector machine-recursive feature elimination (SVM-RFE). The better performance of ATSD-DN suggests its potential for a more complete presentation of time-series changes and effective extraction of early warning information.

Metabolomics is an important branch of systems biology that studies the changes in holistic endogenous metabolites in response to physiological and pathological disturbances^1^,^2^. In the study of disease, metabolomics has shown great potential for exploring the mechanisms of diseases and discovering metabolic biomarkers^3^,^4^,^5^,^6^,^7^. Given that the process of metabolism changes dynamically, monitoring the dynamic responses of metabolites during disease development has attracted increasing interest in recent years.

Dynamic metabolomics studies based on time-series data could possibly provide insight into the interfacial stage between normal states and diseases and further facilitate the screening of biomarkers for early diagnosis. However, optional data processing methods for complex metabolomics time-series data are limited. Metabolomics time-series data are tri-dimensional with a small number of samples, a large amount of features and limited time points^8^,^9^. These characteristics bring difficulties to statistical analysis. Thus, the development of efficient methods to analyze metabolomics time-series data is urgently needed.

To extract effective information from dynamic data, some two-way analysis methods have been used in previous studies, such as principal component analysis (PCA)^10^, partial least squares discriminant analysis (PLS-DA)^11^ and support vector machine-recursive feature elimination (SVM-RFE). However, some important information may be missed due to a lack of information regarding the dynamic properties of these methods^12^, and they simply treat the time course data as a bi-dimensional problem instead of using time-related variation explicitly. This shortcoming has been recognized, and some improved algorithms were proposed to extract more information from time-series data in metabolomics studies. Smilde *et al*.^13^ combined analysis of variance (ANOVA) and simultaneous component analysis to study the variation caused by different factors such as time, doses or combinations, and then proposed ANOVA-simultaneous component analysis (ASCA) method to deal with time course problems. Nueda gave a time-series feature selection technique by calculating the leverage and the squared prediction error based on the ASCA model^14^. Tai *et al*. proposed multivariate empirical Bayes statistical time-series analysis (MEBA) method to rank the features by calculating the Hotelling’s T^2^ 15. Berk *et al*.^8^ used smoothing splines mixed effects (SME) and an associated statistic functional test to detect the features with differences between groups. Subsequently, some data analysis platforms also have been established^16^,^17^ to facilitate the study of time-series data. In our previous work^18^, we also proposed a weighted relative difference accumulation algorithm (wRDA) in which an adapted weight was assigned to every time point for extracting early information regarding complicated diseases. These dynamic methods worked successfully in metabolomics, however, all of them only considered individual metabolites without taking feature association into consideration.

Biological processes are intricate and the relationships among features (such as genes, metabolites and proteins)^19^,^20^,^21^,^22^ are complicated and evolve with dynamic physiological processes. Thus, analyzing data from the perspective of networks could provide more information to understand the associations among features and discover important markers. Fang *et al*.^23^ calculated the information gain (IG) of a ratio between two genes to construct a network. The genes with the largest degrees were regarded as the important factors related to lung cancer. Netzer *et al*.^24^ also constructed a ratio network to select the nodes as biomarkers. If the ratio indicated a statistically significant difference between the classes (e.g., control and obesity groups), then there was an edge between the two corresponding features. Zuo *et al*.^25^ used a low order partial correlation that could reduce spurious edges to infer the network. It is worth noting that most network methods were applied to find key information in static-omics data that discriminated between the different groups, rather than the tracking of features with dynamic differential changes.

In this study, a novel strategy for analyzing time-series data based on dynamic networks (ATSD-DN) in a systematic time dimension was developed. The non-overlapping ratio (NOR) was introduced to quantify the changes in feature ratios with the process of disease development, and provide a novel basis for network construction. Given that the ratio of two metabolites can be assumed to be the result of pathway reactions in which one metabolite is converted into another via single or multiple reaction pathways^26^, ATSD-DN constructed the networks based on the NOR changes of feature ratios along time points, which would facilitate the reflection of physiological or pathological changes. Dynamic concentration analysis and topological structure analysis were performed to analyze the networks and extract early warning information for the disease.

Hepatocellular carcinoma (HCC) is one of the most lethal malignancies^27^, and liver cirrhosis is the major precancerous lesion in the majority of HCC cases^28^. However, until now, early detection of HCC has been a great challenge, especially for the discrimination of precancerous cirrhosis and small malignant HCCs^29^,^30^. Developing new effective methods for the discovery of new biomarkers for early warning of HCC is urgently needed. Due to similarities with histological and genetic features of patients, a diethylnitrosamine (DEN)-induced HCC model can be used to imitate the process of stepwise hepatocarcinogenesis^31^,^32^,^33^. Considering the important role of the liver in ensuring the homeostasis of lipids^11^,^34^, delineating the changes in lipid metabolism would be useful to provide unique insight into early hepatocarcinogenesis and identify novel diagnostic targets. Therefore, ATSD-DN was applied to the time-series lipid data from a rat HCC model induced by DEN administration to define the potential lipid biomarkers for early diagnosis of HCC and validate the performance of ATSD-DN.

## Results

**Figure 1:**
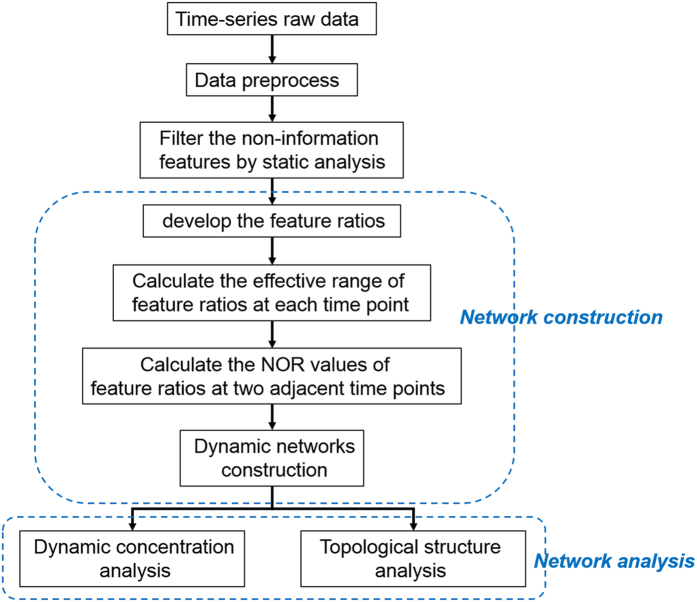
The workflow of ATSD-DN.

The workflow of the ATSD-DN strategy is given in Fig. 1. After filtering the non-informative features by static analysis, ATSD-DN constructed the networks. ATSD-DN provides two techniques: dynamic concentration analysis and topological structure analysis, each of the two network analysis techniques was performed independently to define the informative feature ratios. The PCA score plots based on the feature ratios defined by each network analysis technique alone were used to show the performance of each technique. Finally, the common feature ratios defined by both two techniques were selected and the corresponding performance analysis was also given.

### The construction of dynamic networks

Time-series lipidomics data were analyzed to depict changes in lipid metabolism regarding the process of stepwise hepatocarcinogenesis. A histological examination confirmed that the DEN-induced hepatocarcinogenesis model was successfully produced in this study. The serial progression of hepatocarcinogenesis was divided into three stages: week 8 (hepatitis (H) stage, *T*_1_), weeks 10–14 (cirrhosis (CIR) stage, *T*_2_–*T*_4_) and weeks 16–20 (HCC stage, *T*_5_–*T*_7_). The last week of each stage (i.e., *T*_1_, *T*_4_ and *T*_7_) was the typical time point of the corresponding liver disease stage, while the first weeks of the latter two stages (i.e., *T*_2_ and *T*_5_) were the interfacial points.

In three sub-problems of classification (H *vs.* CIR, H *vs.* HCC and CIR *vs.* HCC), 38 individual features were selected from the first process of noise filtering (i.e., static analysis) at typical time points by SVM-RFE^35^ (Table S1). The multivariate unsupervised PCA analyses were performed to show the discrimination between HCC (*T*_5_–*T*_7_) and non-HCC (*T*_1_–*T*_4_) samples (i.e., hepatitis and cirrhosis samples). The first two principal components captured 65.1% and 71.1% of the total variation from the PCA models based on original all features and these 38 individual features, respectively (Figure S2A,B).

**Figure 2:**
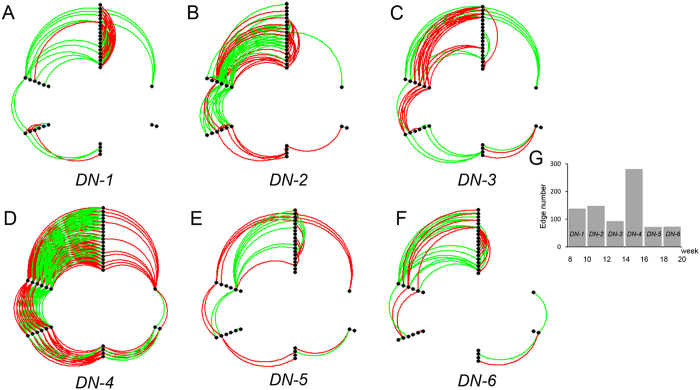
Networks along the time points. (**A–F**) are the dynamic network (*DN*-*i*) based on *T*_*i*_ and *T*_*i*+1_ (*DN*-*i*, 1 ≤ *i* ≤ 6), indicating the dynamic changes in feature ratios during the process of disease progression. (**G**) shows the edge number of each network *DN*-*i* in (**A–F**).

Subsequently, a total of 703 feature ratios were developed based on these 38 individual lipids. For each feature ratio, if the NOR value at two adjacent time points was greater than or equal to 0.85, the corresponding two individual lipids were linked with a red edge. If the NOR was less than or equal to −0.85, the edge was green. As only two time points were considered in each network construction and each time point had exactly the same samples, the sample probability *p*_*t*_ was 0.5. Figure 2 shows the six networks along the 7 time points. In particular, each network can illustrate the changes in feature ratios at two continuous time points, instead of quantification at a single time point.

### Dynamic concentration and topological structure analyses

**Figure 3:**
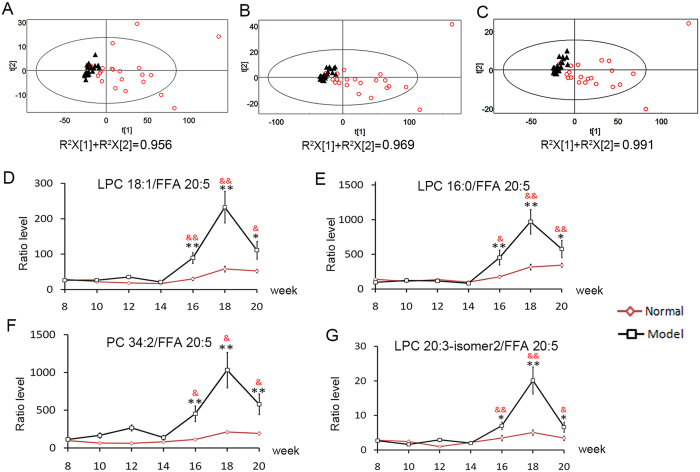
The results of the dynamic concentration analysis and topological structure analysis. (**A,B**) are PCA score plots based on the results of dynamic concentration and topological structure analyses, respectively. (**C**) is PCA score plot based on 15 feature ratios selected by both dynamic concentration and topological structure analyses (Table S3). Non-HCC (black ▲), HCC (red ○). (**D–G**) are the metabolic trajectories (mean ± S.E) of LPC 18:1/FFA 20:5, LPC 16:0/FFA 20:5, PC 34:2/FFA 20:5 and LPC 20:3-isomer2/FFA 20:5 in the discovery set. The black *indicates statistical significance between the control group and model group. The red & indicates statistical significance between the typical CIR (*T*_4_, week 14) and anytime points at the HCC stage (*T*_5_ − *T*_7_, weeks 16–20). *and ^&^:*p* < 0.05, **and ^&&^:*p* < 0.01. LPC, lyso-phosphatidylcholine; PC, phosphatidylcholine; FFA, free fatty acids.

These NOR-based dynamic networks were firstly analyzed from the perspective of dynamic concentration. In Fig. 2, the color of the edges in each network *DN*-*i* indicates the change trend in the effective range for each feature ratio with increased (red) or decreased (green) results at two adjacent time points. To trace the continuous changes of the most important interfacial stage between pre-cancer CIR and early HCC, networks *DN*-4 (*T*_4_–*T*_5_) and *DN*-5 (*T*_5_–*T*_6_) representing the cases in which liver disease developed from pre-cancer cirrhosis to HCC and continued to deteriorate were first emphasized. Therefore, 44 edges with the same color in networks *DN*-4 and *DN*-5 were picked, and the corresponding ratios were retained to construct feature subset 1. The edges with the same colors in *DN-*4 and *DN-*5 represent continuous changes in the dynamics of the circulating metabolites from *T*_4_ to *T*_6_. The PCA analysis was then performed based on the 44 feature ratios to show the discrimination between HCC (*T*_5_–*T*_7_) and non-HCC (*T*_1_–*T*_4_) samples. The score plot shows that the non-HCC and HCC samples could be separated well. A better performance of the PCA model was obtained that 95.6% of the total variation could be explained (Fig. 3A).

In Fig. 2, the dynamics of circulating metabolites could also be analyzed from the perspective of topological structure of networks. In ATSD-DN, the edges between two features represent the dynamics of circulating metabolites over time^26^. Therefore, the network with the most edges among the 6 networks may represent the largest difference in the dynamics of circulating metabolites, which implies physiological or pathological abnormalities. The network with the most edges could be a key stage along the time course and the key point for a particular biological process. The top nodes with the largest degrees in the network would be the key factors signaling the onset of the key stage. For this topological structure analysis, it can be observed that the edge number of network *DN*-4 (*T*_4_–*T*_5_) (Fig. 2G) was the largest among the 6 networks that agreed with the development of HCC validated by the histological examination, indicating activated metabolic disturbance in the interfacial stage between CIR and HCC. Then, the top node with the largest degree (i.e., the number of edges) was chosen. Two nodes (free fatty acid (FFA) 20:5 and triacylglycerol (TAG) 56:9) were observed with the same largest degree in network *DN*-4. It is worth noting that FFA 20:5 was also the top one with the most accumulated degree in 6 networks (Table S2), indicating the continuous metabolic disturbance over time. As a result, 33 ratios associated with FFA 20:5 in network *DN*-4 was retained for subsequent analysis. The separation between non-HCC and HCC stages can also be obviously represented in the PCA score plot based on these 33 feature ratios with 96.9% of the total variation explained (Fig. 3B).

### Definition and external validation of prospective biomarkers

In the discovery set, the common 15 ratios were selected by both dynamic concentration and topological structure analyses (Table S3). In the PCA score plot based on these 15 ratios, the HCC samples could be clearly discriminated from non-HCC subjects with the highest percentage of the total variation explained (i.e., 99.1%; Fig. 3C).

For univariate evaluation, 4 of the 15 ratios showed significant difference between the model and age-matched control groups at the HCC stage (*t*-test, *p* < 0.05) and between *T*_4_ and any time point at the HCC stage (paired *t*-test, *p* < 0.05) simultaneously. Detailed information of these 4 ratio candidates (lyso-phosphatidylcholine (LPC) 16:0/FFA 20:5, LPC 18:1/FFA 20:5, phosphatidylcholine (PC) 34:2/FFA 20:5 and LPC 20:3-isomer2/FFA 20:5) is given in Table 1, and the metabolic trajectories of them are presented in Fig. 3D–G. In the model group, their levels changed slightly at the pre-HCC stage and appeared to increase significantly in the early stage of HCC (*T*_5_). A significant difference between the model and age-matched control groups was also observed at the HCC stage (*T*_5_–*T*_7_). To further illustrate the ability of the 4 feature ratios to discriminate HCC and non-HCC samples, the receiver operating characteristic (ROC) curve was analyzed based on the results for the area under the curve (AUC) and the sensitivity and specificity at the best cut-off points (Table 2). The AUC values of these 4 feature ratios were 0.940–0.980 in the discovery set.

To validate the performances of the 4 biomarker candidates, 36 sera from another 6 model rats with 6 monitoring time points (i.e., *T*_1_–*T*_6_) were analyzed. These 6 rats were sacrificed for histological examination with the validation of HCC at week 18 (*T*_6_). In this external validation set, the AUC values of these 4 candidates were 0.934–0.983 for the discrimination of *T*_1_–*T*_4_ (pre-HCC stage) and *T*_5_–*T*_6_ (HCC stage), confirming the potential of these 4 ratio biomarkers for HCC diagnosis. Considering the similar metabolic characteristics of these 4 candidates and clinical practicability, the feature ratio of LPC 18:1/FFA 20:5 was found to be the potential biomarker with the best AUC value for discrimination. The chromatograms and MS/MS data for LPC 18:1 and FFA 20:5 are provided in Figure S4.

### Comparison with previous methods

To further evaluate the performance of ATSD-DN, this novel approach was compared with two time-series methods wRDA and MEBA, and a popular two-way technique SVM-RFE. The features with the top AUC values in the discrimination of HCC and non-HCC were retained from each method. Phosphatidylinositol (PI) 36:3 was selected by both wRDA and MEBA and TAG 56:8 was selected by SVM-RFE.

**Figure 4:**
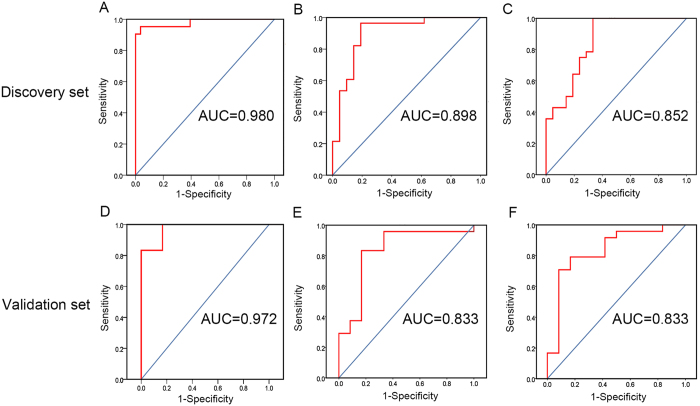
Comparison among ATSD-DN, SVM-RFE, wRDA and MEBA. (**A**,**C**) are ROC curves based on the analysis of ATSD-DN and SVM-RFE in the discovery set, while (**D**,**F**) are the corresponding ROC curves in the validation set. (**B**) is the ROC curve from wRDA and MEBA with the same screening result in the discovery set, and (**E**) is the corresponding ROC curve in the validation set.

In the discovery set, 95.2% of HCC and 96.4% of non-HCC samples could be correctly diagnosed at the best cutoff value based on the results of ATSD-DN (i.e., LPC 18:1/FFA 20:5; Table 2). The AUC value of LPC 18:1/FFA 20:5 was 0.980, which was better than 0.898 of PI 36:3 defined by both wRDA and MEBA and 0.852 of TAG 56:8 defined by SVM-RFE (Fig. 4A–C). Similar comparison results in the validation set are also presented in Fig. 4D–F (the corresponding AUC values were 0.972, 0.833 and 0.833, respectively). The better performance of ATSD-DN may suggest its potential for a more complete presentation of time-series changes.

## Discussion

HCC is one of the most prevalent malignancies with a high mortality rate^27^. Early diagnosis could greatly improve the survival rate^36^. However, unapparent early symptoms and individual differences bring difficulties to early discrimination and seasonable treatment of HCC. Although ultrasonography and some typical tumor markers (e.g., *α*-fetoprotein) have been applied for clinical diagnosis and achieved some successes, they are far from ideal, with high false negative rates^29^,^30^. Developing new efficient methods such as discovering new biomarkers for the early screening of high risk populations is challenging and urgent. Dynamic metabolomics studies based on time-series data can trace the interfacial stage between pre-cancer cirrhosis and HCC and then facilitate the screening of biomarkers for early diagnosis.

To identify the early warning signals of disease deterioration, a new strategy for analyzing time-series data based on dynamic networks in a systematic time dimension was proposed and applied in a prospective cohort study using a diethylnitrosamine (DEN)-induced rat hepatocarcinogenesis model. In this study, noise and irrelevant features were first removed based on the pre-screen. Then, the feature ratio of each of two individual metabolites was developed. The change in the effective range for each feature ratio at two adjacent time points was depicted by the NOR value, which provided the novel basis for network construction. Then, these dynamic networks were used to trace and define the feature ratios with continuous differential changes from two different methods.

In this time-series dataset, to trace the continuous changes of the interfacial stage between CIR and HCC, the networks *DN*-4 and *DN*-5 inferred by *T*_4_, *T*_5_ and *T*_6_ representing the cases in which liver disease developed from pre-cancer cirrhosis to HCC and continued to deteriorate were first emphasized. In Fig. 2, these NOR-based dynamic networks were firstly analyzed from the perspective of dynamic concentration. The edges with the same colors in *DN*-4 and *DN*-5 represent continuous changes in the dynamics of the circulating metabolites from *T*_4_ to *T*_6_, which were picked to facilitate the discrimination between the pre-HCC and HCC stages. Moreover, it is known that there usually exists a key point in disease development that warns the deterioration of the disease. The discovery of this key point and related key information are of great importance to study the disease. In Fig. 2, another perspective of topological structure analysis for these NOR-based dynamic networks showed that network *DN*-4 (*T*_4_–*T*_5_) was the key transition along 7 time points based on the comparison of edge numbers. The discovery demonstrates the validity of the approach that agreed with the development of HCC validated by the histological examination, indicating the activated metabolic disturbance in the interfacial stage between pre-cancer cirrhosis (*T*_4_) and early HCC (*T*_5_).

In the discovery set, four feature ratios, LPC 18:1/FFA 20:5, LPC 20:3-isomer2/FFA 20:5, LPC 16:0/FFA 20:5 and PC 34:2/FFA 20:5, were defined by both dynamic concentration analysis and topological structure analysis, and validated with significant differences between the model and age-matched control groups at the HCC stage (*t*-test, *p* < 0.05) and between *T*_4_ and any time point at the HCC stage (paired *t*-test, *p* < 0.05). ROC analysis indicated the great potential of these four feature ratios for HCC discrimination (AUC = 0.980 for LPC 18:1/FFA 20:5, 0.976 for LPC 20:3-isomer2/FFA 20:5, 0.968 for LPC 16:0/FFA 20:5 and 0.940 for PC 34:2/FFA 20:5). Furthermore, another batch of sera from the external validation set confirmed the effectiveness of the 4 ratio biomarkers for HCC diagnosis (AUC = 0.972 for LPC 18:1/FFA 20:5, 0.934 for LPC 20:3-isomer2/FFA 20:5, 0.983 LPC 16:0/FFA 20:5 and 0.951 for PC 34:2/FFA 20:5). The feature ratio of LPC 18:1/FFA 20:5 was selected as the potential biomarker for further applications.

Monoglycerophospholipid LPC 18:1 can be formed via the hydrolysis of phosphatidylcholine (PC), which has an important role in cell signaling. FFA 20:5 (i.e., eicosapentaenoic acid) has been previously reported to improve steatohepatitis and inhibit the development of HCC^34^,^37^. The decrease in FFA 20:5 may indicate the risk of HCC. In this study, the combination of these two lipids using the biomarker pattern of the LPC 18:1/FFA 20:5 ratio was employed to improve the diagnostic performance. This ratio biomarker pattern would facilitate the magnification of metabolic differences for discrimination. Moreover, compared with traditional individual features or the combination of metabolites from a single pathway, this combination pattern reflects the imbalance of the lipid network from different perspectives of physiology, which would be more informative and robust for HCC risk assessment^38^. Further validation is still needed with a larger cohort of specimens.

To evaluate the efficacy of this new strategy, ATSD-DN was further compared with previous methods (wRDA, MEBA and SVM-RFE). As shown in Fig. 4, the ratio biomarker from ATSD-DN fulfills the best discrimination of HCC and non-HCC samples with the best AUC values in both discovery and validation sets. Based on the comparison results, the better performance of ATSD-DN suggests its great potential for the extraction of early warning information. The advantages of ATSD-DN are as follows: i) this novel strategy is better for the more complete presentation of time-series changes. Rather than screening differentially expressed variables at isolated time points, as in two-way analysis methods, ATSD-DN can be used to trace and define feature ratios with continuous differential changes in a systematic time dimension. ii) The introduction of NOR based on the repeated time series measure facilitates the quantification of changes at two continuous time points and provides a novel basis for network construction. Thus, each network in ATSD-DN presents changes in feature ratios at two continuous time points, which could better reflect the physiological and pathological changes. iii) ATSD-DN analyzes data from the perspective of networks which could possibly provide the insight into the complicated interplay of multiple molecules and be better to explore the development of diseases. Two ways of dynamic concentration and topological structure analyses can be flexibly selected to define the early warning information. iv) ATSD-DN is a data-driven learning method in which few parameters need to be set by the researchers.

It should be noticed that ATSD-DN traces the effective range of a feature ratio along the time points to examine the changes in the feature relationships, and time series repeated measures has been considered in the construction of network. Different from other time-series methods such as ASCA which explores the contributions of different factors or multi-factors, ATSD-DN aims to analyze the networks and extract early warning information for the disease by dynamic concentration analysis and topological structure analysis. In the analysis of metabolomics data, ATSD-DN focuses on the relationship of features to extract the early warning information, and it may ignore some metabolites which associate with the disease but have little relationship with others. Besides, it should be noticed that the present study based on the lipidomics analysis may drop some metabolites which their associate metabolites cannot be detected by the MS. The novel strategy which can combine the feature associations and independent features together should be further developed.

In summary, ATSD-DN analyzes the time-series data from the perspective of networks to define the early warning biomarkers of complicated diseases. The application of ATSD-DN to the rat HCC metabolomics data demonstrated that it is an effective method for identifying potential metabolic biomarkers for early diagnosis. To improve the performance of early risk identification, more construction methods for dynamical networks can be employed in further studies.

## Methods

To study the development of a disease and identify the early warning signals, both control and model samples were collected. Let C denote the control group, M denote the model group and *T*_*i*_ denote a time point, 1 ≤ *i* ≤ *N*, where *N* is the number of time points. Usually, as time goes on, the model samples may suggest different stages of the disease. Let *N*_*s*_ denote the number of the different disease stages along *N* time points.

ATSD-DN defines the prospective information of the disease deterioration based on the dynamic analysis of the networks along the time course. However, not all the features in the metabolic spectrum are involved in the network analysis. Non-informative features are filtered out by static analysis before network construction. ATSD-DN provides two independent techniques to identify the features of interest from the networks. Figure 1 shows the procedure for ATSD-DN.

### Static analysis

It is known that noise and irrelevant features are two factors affecting the efficient analysis of metabolomics data. Given that the model samples experience *N*_*s*_ different biological stages, the features containing little discriminative information from each two-stage segment are noise or unrelated to the problem and should be removed. Thus, ATSD-DN separates the problem into *N*_*s*_(*N*_*s*_ − 1)/2 binary sub-problems and selects the features with discriminative information for each sub-problem to construct the networks for further analysis.

### Network construction

Let *F* = {*f*_1_*, f*_2_*, …, f*_*m*_} be the feature set and *m* be the number of the features. Then, *f*_*it*_ (1 ≤ *i* ≤ *m*, 1 ≤ *t* ≤ *N*) indicates feature *f*_*i*_ at time point *T*_*t*_. Let feature ratio *r*_*ijt*_ = *f*_*it*_/*f*_*jt*_, 1 ≤ *i* < *j* ≤ *m*. A change in *r*_*ijt*_ at the adjacent time points could reflect a change in the biological procedure. Thus, ATSD-DN traces the effective range of a feature ratio along the time points to examine the changes in the feature relationships. The effective range of *r*_*ijt*_ is defined as follows^39^:


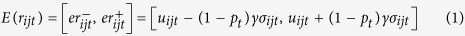


where 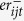 and 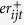 are the floor and the ceiling of the effective range of *r*_*ijt*_. *p*_*t*_ is the sample probability at time point *T*_*t*_ in the corresponding network construction. For the effective range containing least two-thirds of the samples, *γ* is calculated as 1.732 according to Chebyshev Inequality^39^. The variables *u*_*ijt*_ and *σ*_*ijt*_ are the mean and standard deviation of *r*_*ijt*_, and the definitions are as follows:


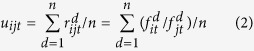



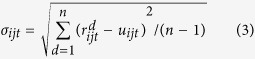


where *n* is the number of the repeated time-series measures, 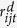 is the value of feature ratio *r*_*ijt*_ at sample (or time-series) *d* (*d* = 1, 2, …, *n*), 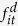 is the value of feature *f*_*i*_ at time point *T*_*t*_ on sample (or time-series) *d* (*d* = 1, 2, …, *n*). For a change in the effective range of a feature ratio between two time points, there exist three cases (Figure S3). In the third case, the effective range of the feature ratio at one time point is included in the effective range at another time point (Figure S3C). This is far from ideal to illustrate the changes in the assumed pathway reactions related to the disease development. Therefore, only the first two cases (Figure S3A,B) are examined in ATSD-DN. Additionally, the changes in the effective range of the feature ratio at the adjacent time points *T*_*t*_ and *T*_*t*+1_ (1 ≤ *t* < *N*) are depicted by the non-overlapping ratio (NOR), which is defined as follows:


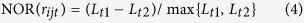


where L_*t*1_ = 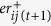 − 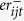 and L_*t*2_ = 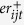 − 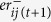. If |NOR(*r*_*ijt*_)| is large, it indicates that the feature ratio *r*_*ijt*_from time *T*_*t*_ to time *T*_*t*+1_ changes greatly, suggesting the continuous metabolic disturbance for the assumed reaction between individual feature *f*_*i*_ and *f*_*j*_. Thus, a network *DN*-*t* could be built based on *T*_*t*_ and *T*_*t*+1_. The network is presented using the rational visualization method of hive plots which is accessed at http://www.hiveplot.net/. Let the features be the vertices of *DN*-*t*. For every pair of features *f*_*i*_ and *f*_*j*_, if |NOR(*r*_*ijt*_)| ≥ *τ*, then there is an edge between *f*_*i*_ and *f*_*j*_ in *DN*-*t*. NOR could also tell the direction of the feature ratio change. NOR(*r*_*ijt*_) > 0 represents the feature ratio *r*_*ijt*_ increasing along two adjacent time points, and NOR(*r*_*ijt*_) < 0 *r*epresents *r*_*ijt*_ decreasing. For simplicity, if NOR(*r*_*ijt*_) ≥ *τ*, the edge between *f*_*i*_ and *f*_*j*_ in *DN*-*t* is colored red, and if NOR(*r*_*ijt*_) ≤ −*τ*, the edge is colored green. If the edge between the two individual features stays red (or green) in consecutive networks, it implies that the feature ratio of these two individual features increases (or decreases) continually along the time points.

### Network analysis

To define the prospective information for a complex disease, ATSD-DN analyzes the networks from two perspectives: dynamic concentration analysis and topological structure analysis.

#### Dynamic concentration analysis

Dynamic concentration analysis investigates the changes in the feature ratios during the course of disease development. As a biological process is always in motion, some signals must exist before a specific time point in a complex disease, such as a malignant tumor. To identify the signals, ATSD-DN focuses on certain time points (without loss of generality, it is assumed to be *N*_*e*_ (0 < *N*_*e*_ < *N*) time points) before the typical time point *T*_*s*_ (1 < *s* ≤ *N*) of the disease. If the effective range of the ratio between the features along *N*_*e*_ time points continues to change in the same direction (such as continuous increasing or decreasing), it indicates a continuous metabolic disturbance. Therefore, to identify the early warning signal for the specific time point of disease, the networks *DN*-*i* (*s* − *N*_*e*_ ≤ *i* < *s* − 1) are examined, and the edges that remain the same color in *DN*-*i* are selected. The corresponding ratios are selected as the signals of the specific time point of the disease and constitute feature subset 1.

#### Topological structure analysis

The topological structures of the *N*-1 networks along *N* time points can also indicate the biological changes over time. If the edge number of *DN*-*t* (1 ≤ *t* < *N*) is large, it implies that many pathway reactions experience large changes in the reaction rate and the organism experiences a relatively drastic biological change. Thus, *DN*-*t* (1 ≤ *t* < *N*) with the most edges could be a key stage along the time course and may be the key point for a particular biological process. The nodes with the largest degrees in the network would be the key factors signaling the onset of the key stage. Thus, in topological structure analysis, ATSD-DN analyzes the edge numbers of *N*-1 networks along *N* time points and focuses on the one (*DN*-*t*, 1 ≤ *t* < *N*) that has the most edges. It ranks the nodes in *DN*-*t* according to their degrees in a descending order, and the top *k* ≥ 1 nodes are selected and the feature ratios corresponding to the edges associated with the *k* nodes are selected to constitute feature subset 2.

Each of the two network analysis techniques has its own merits for extracting early warning information. Therefore, they can be used flexibly to analyze the time-series data and to define the potential biomarkers independently. It is also possible to use them simultaneously to get the feature subset by union or intersection of feature subset 1 and feature subset 2.

### The application of ATSD-DN to metabolomics data from a rat HCC model

ATSD-DN was applied to the time-series data to define the potential biomarkers for early diagnosis of HCC. The data include a discovery set and a validation set. ATSD-DN was performed on the discovery set to identify prospective information. The validation set was used to test the results of ATSD-DN on the discovery set.

#### Time-series data source

In this study, time-series data were obtained from the animal model with DEN-induced stepwise hepatocarcinogenesis. This animal experiment was conducted at the experimental animal center of Dalian Medical University (Dalian, China), in compliance with national guidelines for the care and use of laboratory animals. The study protocol was reviewed and approved by the institutional reviewer board of Dalian Medical University, Dalian, China. And the experiment was carried out in accordance with the approved guidelines.

This rat model has been described detailedly in our previous report^11^,^40^. Briefly, a total of 55 male Sprague-Dawley (S.D.) rats were enrolled in the present study at the age of 42 days (i.e., week 0). Then, after two weeks of adaptation, all rats were randomly divided into control (*n* = 10) and model (*n* = 45) groups, administrated with saline and DEN at 70 mg/kg body weight respectively via intraperitoneal injection. The injection was performed once a week between week 2 and week 11, and 14 rats from the model group died during the administration.

Histological examination was performed to monitor the progress of stepwise hepatocarcinogenesis based on the sacrifice of model rats, until all of the surviving animals (*n* = 10 for control and *n* = 7 for model groups) were finally sacrificed in week 20. Collected liver tissues were fixed in 10% buffered formalin and embedded in paraffin for histological examination, which confirmed that the DEN-induced hepatocarcinogenesis model was successfully produced in the present study.

The collection of time-series sera set was conducted from week 8 to week 20 once every 2 weeks (i.e., 7 monitoring time points). The discovery data included 10 rats from the control group and 7 rats from the model group. A total of 119 time-series sera were then collected from all 7 monitoring time points once every two weeks from week 8 to week 20. Thus, the number of the time points for the discovery set was 7; i.e., *N* = 7. In the model group, the first time point *T*_1_ was week 8 (M8) and the 7th time point *T*_7_ was week 20 (M20). Similarly, C8 and C20 were week 8 and week 20 in the control group.

Furthermore, 36 sera from another 6 model rats were used for validation. These 6 rats were sacrificed for histological examination with the affirmance of HCC at week 18. Therefore, their sera were collected from 6 monitoring time points (i.e., *T*_1_–*T*_6_).

#### Profiling of lipids by LC-MS analysis

Time-series serum samples were analyzed to perform a non-targeted lipidomics study using an ACQUITY ultra-performance liquid chromatography (UPLC) system (Waters, USA) coupled with a tripleTOF™ 5600 plus mass spectrometer (AB Sciex, USA). Details regarding lipidomics analysis including serum preparation and instrument methods are provided in the Supplemental Information.

#### Data analysis

Based on the accurate *m*/*z*, retention behavior and MS/MS fragmentation pattern, lipid species were first identified with LipidView and PeakView software (AB Sciex, USA). Then, the quantitative information for detected lipids was extracted using MultiQuan software (AB Sciex, USA) with a mass width of ± 0.01 Da and retention time width of ± 0.15 min. Before statistical analysis, the relative abundance of all lipids was calculated by normalizing to the area of corresponding internal standards. Finally, a time-series dataset was exported to the ATSD-DN strategy.

Seven time points include three different stages of liver disease (*N*_*s*_ = 3): hepatitis, cirrhosis and hepatocellular carcinoma. The features containing little discriminative information for every two-stage segment were removed. SVM-RFE was first applied on three binary sub-problems (H *vs.* CIR, H *vs.* HCC, CIR *vs.* HCC). Five-fold cross-validation was run fifty times for each sub-problem. In SVM-RFE, the kernel function and penalty factor were set as the *liner* kernel function and 1, respectively. The implementation of SVM was performed with LIBSVM (available at http://www.csie.ntu.edu.tw/~cjlin/libsvm). MEBA was from http://www.metaboanalyst.ca/faces/Secure/upload/TimeUploadView.xhtml. All the algorithms were written in C++.

The selected feature subsets of the three sub-problems were united and used to infer the networks with τ = 0.85. *T*_7_ is the typical HCC stage and *T*_4_ is the typical CIR stage. It is known that HCC usually develops from CIR. Thus, *N*_*e*_ = 3 time points before typical HCC (*T*_*s*_ = 7) were studied to define the early warning information of for HCC by means of dynamic concentration analysis. Thus, *DN*-4 and *DN*-5 were inferred by these three time points. The feature ratios corresponding to the edges whose colors stay the same in *DN*-4 and *DN*-5 were selected to constitute feature subset 1.

The edge numbers of the 6 networks along the 7 time points were analyzed. The network that had the greatest number of edges was selected. Its nodes were ranked according to their degrees in descending order, and the top ranked node was selected. The ratios corresponding to the edges linked with the top ranked node were selected to constitute feature subset 2.

### The compared methods

#### wRDA

The mean value and standard deviation were used to measure the differences for a feature between the control and model groups^18^. An adapted weight was assigned to each time point for extracting early information on complicated diseases. Subsequently, a false discovery rate (FDR)^41^ was used to evaluate the selected feature subset. The lower the FDR, the better the selected features. In this study, the weights of non-HCC and HCC stages were 0.1 and 0.2, respectively. The top 30 features with the largest scores with FDR = 0% were constructed as the final feature subset.

#### MEBA

A time-course analysis method based on multivariate empirical Bayes statistical which could evaluate the importance of the features by the Hotelling’s T^2^ 15. The top 30 features with the largest Hotelling’s T^2^ were constructed as the final feature subset.

#### SVM-RFE

This method has been widely applied to select discriminative features from the high-dimensional metabolomics data^35^,^42^,^43^,^44^,^45^,^46^. It removes the least important features iteratively. In each iteration, the weight of each feature in the current feature subset is re-measured based on the contribution to the hyper-plane, and *r*% features with the smallest weights are removed. This process is repeated until the current feature subset is empty. The feature subset with the largest accuracy rate in the iteration is kept as the selected features subset.

## Additional Information

**How to cite this article**: Huang, X. *et al*. A New Strategy for Analyzing Time-Series Data Using Dynamic Networks: Identifying Prospective Biomarkers of Hepatocellular Carcinoma. *Sci. Rep.* **6**, 32448; doi: 10.1038/srep32448 (2016).

## Supplementary Material

Supplementary Information

## Figures and Tables

**Table 1:** Prospective ratio biomarkers selected by ATSD-DN.

Lipids 1 (Numerator)	Mode	m/z	m/z error (ppm)	t^R^ (min)	Lipids 2 (Denominator)	Mode	m/z	m/z error (ppm)	t^R^ (min)	*p* value					
										C16 *vs*. M16	C18 *vs*. M18	C20 *vs*. M20	M14 *vs*. M16	M14 *vs*. M18	M14 *vs*. M20
LPC 16:0	Pos	496.3398	3	1.71	FFA 20:5	Neg	301.2173	−2.8	1.51	7.25E-03	6.91E-04	4.57E-02	1.59E-02	3.01E-03	7.84E-03
PC 34:2	Pos	758.5695	4	6.84						1.45E-03	7.43E-04	4.28E-03	4.17E-02	1.13E-02	2.43E-02
LPC 18:1	Pos	522.3554	3.6	1.81						6.39E-04	3.68E-04	1.52E-02	9.05E-03	4.09E-03	1.29E-02
LPC 20:3-isomer2	Pos	546.3554	−0.1	1.67						1.81E-02	4.52E-04	4.15E-02	7.96E-03	4.35E-03	1.96E-02

These lipids were identified based on the accurate m/z, retention behavior and MS/MS fragmentation pattern.

**Table 2:** The results of ROC analysis.

Feature ratio	Date set	AUC	S.E	Hotelling: 95%		Sensitivity	Specitivity
				Lower	Upper		
LPC 18:1/FFA 20:5	Discovery set	0.980	0.019	0.941	1.000	0.952	0.964
	Validation set	0.972	0.023	0.926	1.000	1.000	0.833
LPC 16:0/FFA 20:5	Discovery set	0.968	0.032	0.905	1.000	0.952	1.000
	Validation set	0.983	0.017	0.950	1.000	1.000	0.875
PC 34:2/FFA 20:5	Discovery set	0.940	0.030	0.881	1.000	0.952	0.750
	Validation set	0.951	0.032	0.889	1.000	1.000	0.833
LPC 20:3–isomer2/ FFA 20:5	Discovery set	0.976	0.017	0.942	1.000	0.905	0.964
	Validation set	0.934	0.044	0.849	1.000	0.833	0.958

ROC, receiver operating characteristic curve; AUC, area under the curve.
