# Supplementary material for: A New Strategy for Analyzing Time-Series Data Using Dynamic Networks: Identifying Prospective Biomarkers of Hepatocellular Carcinoma
Source: Sci Rep. 2016 Aug 31;6:32448. doi: 10.1038/srep32448 (PMC5006023; doi:10.1038/srep32448)
Supplement: Supplementary Information [file srep32448-s1.pdf]

A New Strategy for Analyzing Time-Series Data Using Dynamic Networks: Identifying Prospective Biomarkers of Hepatocellular Carcinoma

Xin Huang[a,♠], Jun Zeng[b,♠], Lina Zhou[b], Chunxiu Hu[b], Peiyuan Yin[b*], Xiaohui Lin[a*]

[a] School of Computer Science & Technology, Dalian University of Technology, 116024 Dalian, China.

[b] Key Laboratory of Separation Science for Analytical Chemistry, Dalian Institute of Chemical Physics, Chinese Academy of Sciences, Dalian 116023, China.

Materials and chemicals

All reagents were HPLC-grade. Methanol (MeOH), acetonitrile (ACN) and isopropyl alcohol (IPA) were purchased from Merck (Germany). Dichloromethane (DCM) was purchased from Spectrum Chemical Mfg. Corp. (USA). Ultrapure water (18.2 MΩ-cm, TOC = 6 ppb) was obtained from a Milli-Q system (Millipore, USA).

Ammonium acetate (AmAc, > = 98%) was purchased from Sigma-Aldrich (USA). Lipid standards of phosphatidylcholine (PC) (19:0/19:0), phosphatidylethanolamine (PE) (17:0/17:0), lyso-phospatidylcholines (LPC) (19:0), sphingomyelin (SM)(d18:1/12:0), triacylglycerol (TAG) (15:0/15:0/15:0), ceramide (CER) (d18:1/17:0), palmitic acid (FFA C16:0)-d3 and stearic acid (FFA C18:0)-d3 were purchased from Avanti Polar Lipids (USA) or Sigma-Aldrich (USA), and used as internal standards.

Serum preparation

To extract lipids, each 50 μL of serum was successively added with 450 μL of

prepared methanol (containing internal standards of 0.856 μM of PC 19:0/19:0, 0.435 μM of PE 17:0/17:0, 0.517 μM of LPC19:0, 0.215 μM of SM d18:1/12:0, 0.653 μM of TAG 15:0/15:0/15:0, 0.569 μM of CER d18:1/17:0, 2.144 μM of FFA C16:0-d3, and 1.933 μM of FFA C18:0-d3), 500 μL of chloroform and 200 μL of Milli-Q water. 1 min of vortex was performed after each addition, and 5 min of standing was conducted after the last vortex. Subsequently, the mixture was centrifuged at 12,000 g for 10 min at 4 °C to form a two-phase system. The lower layer was then transferred, and divided into two aliquot (200 μL for each analysis mode) after the re-centrifugation (15,000 g, 10 °C, 10 min). Samples were lyophilized for store (-80 °C) and reconstituted before analysis.

Quality control (QC) samples were prepared by pooling equal aliquot of each serum sample, followed by lipid extraction as real samples.

Profiling of lipids by LC-MS analysis

Non-targeted lipidomics analysis was performed using an ACQUITY ultra performance liquid chromatography (UPLC) system (Waters, USA) coupled with an tripleTOF™ 5600 plus mass spectrometer (AB Sciex, USA), complying with the method described in our previous report with minor modifications[1].

Briefly, an ACQUITY UPLC BEH C8 column (2.1 × 100 mm, 1.7 μm) (Waters, USA) was used for chromatographic separation with column temperature controlled at 55 °C. The mobile phases were A (ACN:H2O = 6:4, 10 mM ammonium acetate) and B (IPA:ACN = 9:1, 10 mM ammonium acetate), respectively. The gradient elution started with 32% B for 1.5 min, followed by a linearly increase to 85% B from 1.5 to 15.5 min. The mobile phase B was further increased to 97% in 0.1 min, and kept for 2.4 min from 15.6 to 18 min. Finally, mobile phase B was decreased to 32% in the

next 0.1 min, and equilibrated for 1.9 min until the next injection. Then, the elution was maintained for 20 min for each injection with a flow rate of 0.26 mL/min. The sample tray temperature was controlled at 10 °C, and the sample injection volume was 5 μL.

For MS analysis, TOF MS full scan for lipid profiling and information-dependent acquisition (IDA) for MS/MS data were performed simultaneously. When electrospray (ESI) was performed in the positive ion mode, the major parameters were set as follows: ion source gas 1 (Gas1), 50 psi; ion source gas 2 (Gas2), 50 psi; curtain gas (CUR), 35 psi;temperature (TEM), 500 °C; ion spray voltage floating (ISVF), 5500 V; declustering potential (DP), 100V; collision energy (CE) 35 V; collision energy spread (CES), ± 15 V; mass range of full scan, 400-1,200; mass range of IDA, 100-1,200. In the negative mode, the parameters were set as follows: ion source gas 1 (Gas1), 55 psi; ion source gas 2 (Gas2), 55 psi; curtain gas (CUR), 35 psi; temperature (TEM): 600 °C; ion spray voltage floating (ISVF), -4500 V; declustering potential (DP), -100V; collision energy (CE), -45 V; collision energy spread (CES), ± 20 V; mass range of full scan, 90-1,000; mass range of IDA, 90-1,000. For the acquisition of MS/MS information, ions with top 10 intensity were subjected to the high resolution IDA.

Quality evaluation for lipidomics data

To monitor the robustness of lipid profiling, quality control (QC) samples were inserted into the analysis batch in each set of 10 real samples. All real samples were analyzed in random order. Then, the quality of acquired data was evaluated by analyzing the analytical characteristics of QCs. The score plots of unsupervised principal component analysis (PCA) show that the deviation of QCs was within 2

times of standard deviation (SD), demonstrating the statistical acceptance of acquired data (Figure S1A and C). Specifically, the distributions of %RSD for metabolites reveal that in the positive mode, 100% of sum of number had a %RSD of less than 30% (Figure S1B). While the result was 97.65% for negative responses (Figure S1D). The evaluation of analytical characteristics ensured the satisfactory stability and reproducibility of present lipidomics study, and the high quality of acquired data.

To reduce the error, lipids with a %RSD in QCs higher than 30% were discarded from the dataset.

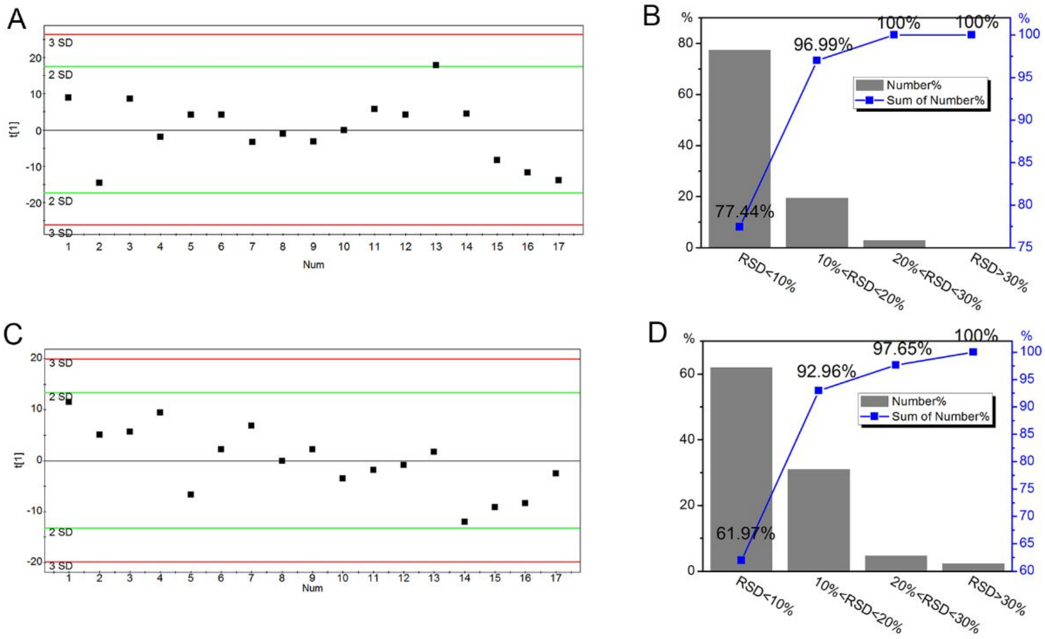

Figure S1. Quality evaluation for lipidomics data. For QC samples, (A) and (C) are PCA score plots for positive and negative mode, respectively. (B) and (D) present the distribution of %RSD for metabolites.

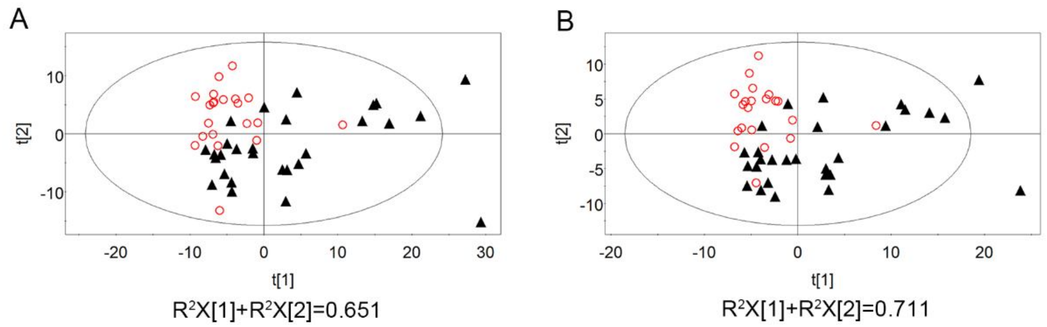

Figure S2. PCA score plots based on origin all features (A) and 38 pre-defined individual features (B; Table S1). Non-HCC (black ▲), HCC (red ○).

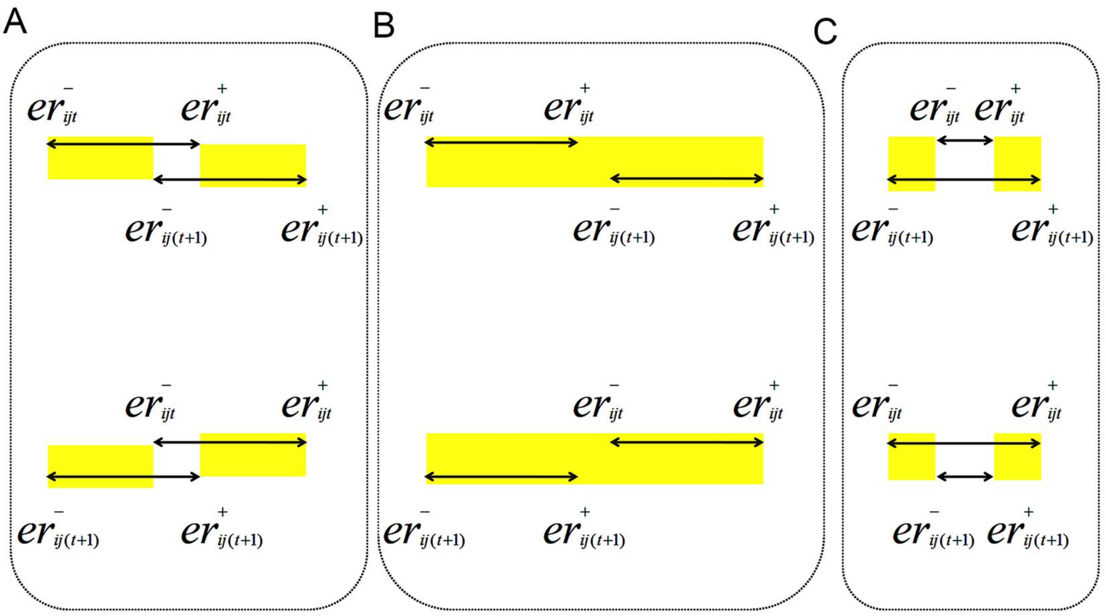

Figure S3. Graphic presentation of the non-overlapping ratio (NOR). (A)–(C) are three NOR cases.

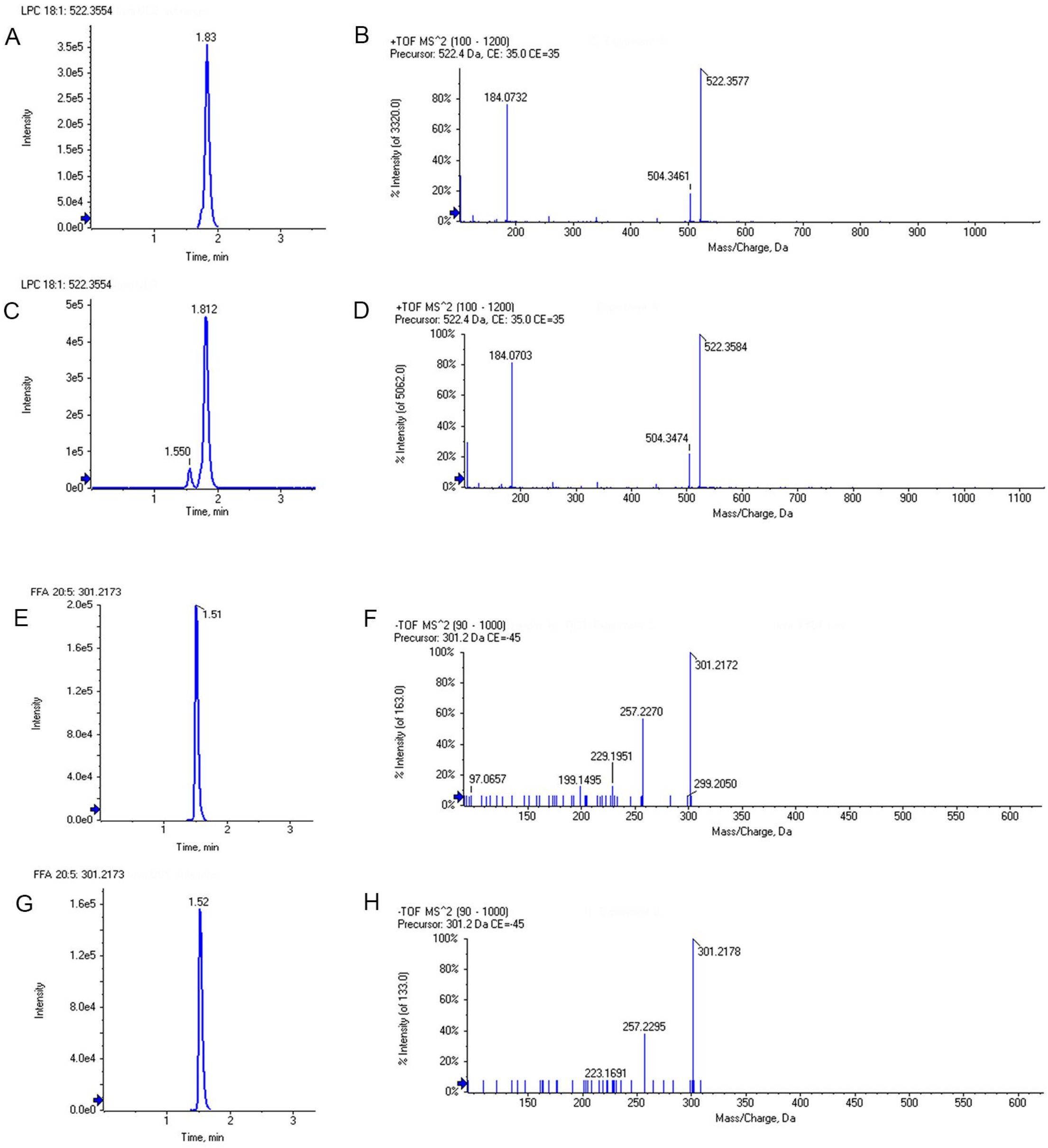

Figure S4. Typical characteristics of LPC 18:1 and FFA 20:5. (A) and (C) are the extracted ion chromatograms of LPC 18:1 for the discovery and validation sets, respectively. (B) and (D) are the MS/MS spectra of LPC 18:1 for the discovery and validation sets, respectively. (E) and (G) are the extracted ion chromatograms of FFA 20:5 for the discovery and validation sets, respectively. (F) and (H) are the MS/MS spectra of FFA 20:5 for the discovery and validation sets, respectively.

Table S1. Pre-defined individual features based on static analysis.

Metabolites	Mode	Existing form	m/z	t_R (min)	C8		M8		C10		M10		C12		M12		C14		M14		C16		M16		C18		M18		C20		M20	
					Average	SD	Average	SD	Average	SD	Average	SD	Average	SD	Average	SD	Average	SD	Average	SD	Average	SD	Average	SD	Average	SD	Average	SD	Average	SD	Average	SD
FFA 18:0	Neg	$[M-H]^-$	283.2642	2.66	5.01	0.83	5.73	0.90	4.96	0.82	5.77	1.29	5.19	0.50	4.73	0.77	4.92	0.54	5.16	0.43	4.59	0.53	4.00	0.59	5.40	0.64	4.77	0.63	5.20	0.56	4.57	0.66
FFA 18:1	Neg	$[M-H]^-$	281.2486	2.16	4.27	1.46	9.01	2.36	5.69	1.88	9.34	3.81	5.96	1.35	5.82	2.06	4.70	1.33	6.34	1.70	4.55	1.17	3.27	1.15	5.66	1.35	3.85	1.36	5.17	1.17	4.22	2.47
FFA 18:2	Neg	$[M-H]^-$	279.2329	1.80	5.72	1.30	9.25	1.94	7.50	1.80	10.09	3.66	7.21	1.53	6.75	2.08	6.27	1.54	7.79	1.90	5.28	1.21	4.09	1.28	6.61	1.34	4.28	1.28	5.53	1.13	4.49	1.40
FFA 20:4	Neg	$[M-H]^-$	303.2329	1.74	3.00	0.36	2.95	0.48	3.19	0.33	3.12	0.59	3.63	0.43	2.02	1.00	2.78	0.53	2.43	0.79	2.85	0.40	2.18	0.61	3.12	0.24	2.23	0.55	3.02	0.43	2.42	0.58
FFA 20:5	Neg	$[M-H]^-$	301.2173	1.51	0.31	0.08	0.40	0.08	0.41	0.17	0.40	0.20	0.29	0.09	0.36	0.09	0.38	0.08	0.51	0.13	0.23	0.08	0.15	0.13	0.14	0.04	0.06	0.03	0.10	0.03	0.11	0.06
FFA 22:6	Neg	$[M-H]^-$	327.2329	1.60	1.47	0.44	1.70	0.32	2.16	0.78	1.89	0.66	1.63	0.50	1.46	0.57	1.43	0.39	1.53	0.34	1.20	0.38	0.78	0.40	1.19	0.31	0.52	0.17	0.73	0.13	0.62	0.22
PI 38:3	Neg	$[M-H]^-$	887.5655	6.43	1.27	0.61	1.80	0.73	1.17	0.37	1.73	0.35	0.66	0.41	2.10	0.52	1.57	0.63	1.51	0.69	1.19	0.50	0.96	0.36	1.08	0.57	0.82	0.20	0.49	0.18	0.73	0.25
PI 38:4	Neg	$[M-H]^-$	885.5498	6.15	20.48	5.27	13.46	3.02	19.96	4.91	17.94	2.58	21.97	8.12	24.01	4.03	23.88	7.31	19.96	4.98	24.57	8.40	21.94	3.01	26.71	4.97	23.46	4.43	24.74	6.80	21.98	3.96
LPC 16:0	Pos	$[M+H]^+$	496.3398	1.71	41.71	4.87	37.87	4.15	40.91	3.97	38.27	6.07	36.62	4.42	40.89	8.15	36.56	3.63	38.84	5.79	35.98	4.65	43.10	6.20	39.12	3.52	48.58	7.78	31.83	3.67	45.85	6.97
LPC 18:1	Pos	$[M+H]^+$	522.3554	1.81	8.64	1.37	10.02	0.64	7.88	1.56	8.70	2.01	5.03	1.07	12.17	3.02	6.03	1.52	9.92	2.25	6.20	1.66	9.54	2.66	7.26	2.29	11.61	1.30	4.83	0.80	8.99	2.06
LPC 20:3-isomer2	Pos	$[M+H]^+$	546.3554	1.67	0.89	0.32	1.04	0.12	0.90	0.36	0.59	0.24	0.28	0.14	1.02	0.43	0.81	0.45	0.92	0.21	0.71	0.41	0.84	0.38	0.67	0.41	1.01	0.15	0.32	0.17	0.57	0.21
LPC 20:4	Pos	$[M+H]^+$	544.3398	1.53	8.86	1.49	7.23	0.87	9.16	2.74	5.45	1.68	12.06	2.47	8.76	3.49	10.55	2.61	9.47	2.71	12.55	2.77	10.77	3.41	13.58	2.32	14.28	3.35	13.90	2.26	11.00	2.87
LPC 22:6	Pos	$[M+H]^+$	568.3398	1.47	1.56	0.45	2.23	0.16	1.67	0.46	1.57	0.60	1.43	0.35	2.73	1.00	1.63	0.45	2.60	0.63	1.70	0.53	2.26	0.66	1.50	0.39	2.15	0.59	1.32	0.35	1.56	0.54
PC 34:2	Pos	$[M+H]^+$	758.5695	6.84	29.73	8.64	44.54	8.19	24.37	5.95	52.76	12.38	17.47	6.62	91.01	25.98	28.99	9.25	64.76	25.94	23.54	7.20	47.73	18.28	27.09	8.62	50.57	3.66	17.85	4.35	45.01	7.07
PC 36:2	Pos	$[M+H]^+$	786.6008	7.71	19.56	6.04	23.71	5.10	17.79	3.96	26.64	5.48	9.92	4.30	47.94	15.99	19.52	6.84	32.98	15.62	16.16	5.70	21.74	10.38	17.29	7.45	21.60	2.12	9.94	2.31	19.88	3.24
PC 36:3	Pos	$[M+H]^+$	784.5851	7.00	6.33	1.26	9.90	1.99	5.26	0.92	10.17	3.05	2.01	0.82	18.22	5.92	4.75	2.08	12.80	6.53	3.86	1.57	6.82	4.13	3.93	1.86	7.39	1.35	1.96	0.66	6.41	2.56
PC 36:4-2	Pos	$[M+H]^+$	782.5695	6.79	9.10	2.27	11.05	1.20	9.80	1.93	12.81	1.53	14.13	5.23	20.86	4.24	14.70	5.36	16.76	3.41	18.29	3.40	18.75	2.28	21.14	4.34	21.63	6.00	20.05	3.43	21.35	4.69
PC 36:5-2	Pos	$[M+H]^+$	780.5538	6.20	0.55	0.25	0.85	0.16	0.54	0.19	0.89	0.40	0.29	0.13	2.25	0.83	0.81	0.39	1.88	0.97	0.54	0.18	0.76	0.65	0.40	0.19	0.58	0.17	0.27	0.07	0.68	0.24
PC 38:3	Pos	$[M+H]^+$	812.6164	8.00	1.07	0.45	1.51	0.40	1.16	0.35	1.24	0.25	0.50	0.32	1.52	0.30	1.24	0.53	1.05	0.35	1.01	0.40	0.79	0.32	0.95	0.55	0.66	0.10	0.38	0.14	0.58	0.11
PC 38:4	Pos	$[M+H]^+$	810.6008	7.66	11.38	2.92	9.16	1.74	12.93	3.25	9.48	0.82	18.97	8.09	15.52	2.96	19.43	8.07	12.63	3.05	24.38	5.83	14.32	2.35	28.87	7.94	15.15	4.44	26.95	4.21	16.77	3.92
SM 34:1;2	Pos	$[M+H]^+$	703.5749	6.46	15.78	3.84	12.21	2.14	15.16	2.85	18.30	4.63	16.01	5.93	25.86	5.79	21.99	6.28	19.85	3.78	19.25	6.45	18.24	3.06	20.82	5.85	18.48	2.88	18.99	5.78	18.60	3.36
SM 34:2;2	Pos	$[M+H]^+$	701.5592	5.68	1.43	0.35	1.44	0.31	1.35	0.30	1.80	0.48	1.46	0.52	2.00	0.36	1.90	0.60	1.60	0.29	1.63	0.60	1.45	0.25	1.63	0.47	1.35	0.25	1.46	0.45	1.32	0.24
SM 36:1;2	Pos	$[M+H]^+$	731.6062	7.39	0.70	0.24	1.62	0.42	0.69	0.20	2.74	1.37	1.08	0.42	3.07	1.31	1.34	0.72	1.65	0.44	1.23	0.61	1.72	0.70	1.28	0.49	1.33	0.17	1.56	0.88	1.40	0.21
SM 42:1;2	Pos	$[M+H]^+$	815.7001	9.85	7.64	2.00	5.84	1.28	7.50	1.45	6.05	0.83	7.65	2.48	6.89	1.60	9.89	2.73	7.28	1.74	8.61	2.01	8.16	1.13	9.49	2.41	8.36	1.71	8.41	1.88	7.88	1.72
SM 42:2;2-isomer1	Pos	$[M+H]^+$	813.6844	9.07	9.61	2.66	12.41	2.59	8.87	1.62	13.26	1.57	8.58	3.56	17.96	2.27	11.12	3.58	14.42	3.81	10.34	3.38	12.09	2.12	10.77	3.92	12.14	2.11	8.87	2.39	11.19	2.77
SM 42:2;2-isomer2	Pos	$[M+H]^+$	813.6844	9.30	1.99	0.56	1.91	0.47	1.93	0.43	2.09	0.37	2.08	0.70	2.07	0.41	2.72	0.86	2.13	0.51	2.11	0.74	2.03	0.32	2.00	0.57	1.88	0.38	1.81	0.45	1.79	0.46
SM 42:3;2	Pos	$[M+H]^+$	811.6688	8.43	3.77	1.10	6.28	1.46	3.53	0.70	6.90	1.02	3.39	1.36	8.74	1.77	4.65	1.51	6.81	1.78	3.93	1.28	5.52	0.93	4.31	1.47	5.55	1.07	3.63	0.99	5.10	1.53
TAG 50:1	Pos	$[M+NH_4]^+$	850.7858	12.87	4.12	2.02	2.25	0.75	2.66	1.44	1.66	0.86	0.93	0.44	2.14	1.01	1.69	1.19	1.26	0.44	1.85	1.20	1.32	0.56	2.06	0.83	1.65	0.73	1.19	0.40	2.01	2.42
TAG 52:2	Pos	$[M+NH_4]^+$	876.8014	12.92	10.01	4.29	7.39	2.08	6.73	3.30	5.76	2.96	2.62	1.67	7.02	3.00	4.13	2.54	3.81	1.50	5.34	3.07	4.32	1.82	7.88	3.91	5.96	2.33	4.18	1.58	5.18	5.65
TAG 52:3	Pos	[M+NH4]+	874.7858	12.58	17.29	6.58	9.74	3.19	12.53	5.51	7.94	3.94	5.80	3.97	9.38	5.36	8.62	4.48	5.60	1.45	11.23	6.24	6.49	2.69	17.11	6.54	9.13	4.35	10.22	3.42	8.34	7.86
TAG 54:2	Pos	[M+NH4]+	904.8327	13.29	0.87	0.28	0.68	0.17	0.59	0.29	0.64	0.48	0.23	0.10	0.95	0.72	0.38	0.26	0.41	0.20	0.42	0.30	0.40	0.23	0.44	0.20	0.49	0.28	0.25	0.07	0.50	0.59
TAG 54:3	Pos	[M+NH4]+	902.8171	12.97	3.28	1.01	2.96	0.65	2.19	1.02	2.62	1.39	0.75	0.31	3.85	2.49	1.37	0.93	1.78	0.86	1.57	1.10	1.61	0.85	1.95	0.89	2.23	0.97	1.03	0.32	1.74	1.55
TAG 54:4	Pos	[M+NH4]+	900.8014	12.64	5.74	1.99	4.35	1.02	3.77	1.92	3.73	1.74	0.99	0.42	5.49	4.30	2.41	1.85	2.59	0.98	2.62	1.97	2.37	1.05	3.33	1.58	3.47	1.55	1.67	0.56	2.39	1.54
TAG 54:7-isomer2	Pos	[M+NH4]+	894.7545	11.70	2.07	1.07	0.91	0.33	2.02	0.96	0.80	0.28	1.18	0.88	1.41	1.08	1.87	0.89	1.08	0.19	1.41	0.67	0.47	0.33	1.36	0.73	0.36	0.17	0.79	0.31	0.54	0.32
TAG 56:7	Pos	[M+NH4]+	922.7858	12.26	7.54	2.98	3.76	1.16	6.93	2.25	3.72	1.33	3.34	1.79	5.15	3.03	4.75	1.95	3.56	0.57	4.98	2.34	2.50	0.93	5.61	2.05	2.57	0.95	3.11	1.00	2.54	1.12
TAG 56:8	Pos	[M+NH4]+	920.7701	11.92	7.25	2.63	2.84	1.05	7.38	2.48	3.04	1.17	4.85	2.51	4.58	3.53	6.22	2.34	3.32	0.40	5.99	2.45	1.96	1.02	6.27	2.16	1.59	0.88	3.38	1.18	1.81	0.93
TAG 56:9	Pos	[M+NH4]+	918.7545	11.48	0.84	0.35	0.53	0.14	0.81	0.32	0.56	0.23	0.37	0.17	1.04	1.04	0.69	0.33	0.67	0.18	0.50	0.28	0.26	0.19	0.46	0.21	0.17	0.07	0.27	0.08	0.26	0.16
TAG 58:10	Pos	[M+NH4]+	944.7701	11.60	0.90	0.26	0.71	0.17	0.94	0.30	0.86	0.29	0.74	0.19	1.42	1.27	0.89	0.27	0.87	0.16	0.77	0.34	0.45	0.19	0.78	0.21	0.35	0.13	0.65	0.14	0.52	0.15

These metabolites were selected from the first process of noise filtering (i.e., static analysis).
FFA, free fatty acids; LPC, lyso-phosphatidylcholine; PC, phosphatidylcholine; PI, phosphatidylinositol; SM, sphingomyelin; TAG, triacylglycerol

Table S2. The change of degree in six networks.

Lipids	DN-1	DN-2	DN-3	DN-4	DN-5	DN-6	Sum
FFA 20:5	1	4	25	33	16	8	87
TAG 56:9	2	4	4	33	5	6	54
TAG 54:7-isomer2	2	4	5	29	2	6	48
TAG 58:10	11	4	4	27	6	10	62
FFA 22:6	1	7	7	25	13	3	56
PC 36:5-isomer2	2	22	2	24	1	7	58
FFA 18:1	0	22	7	20	0	0	49
PC 36:4-isomer2	9	11	2	20	2	1	45
TAG 56:8	5	3	4	20	5	1	38
FFA 18:2	0	25	10	19	0	0	54
PI 38:4	15	8	1	19	0	2	45
PC 38:4	5	13	3	18	1	2	42
PC 36:3	4	6	1	17	3	2	33
TAG 52:2	8	2	11	15	8	4	48
TAG 56:7	7	5	4	14	6	4	40
LPC 16:0	4	7	0	13	2	3	29
SM 34:2;2	10	9	2	13	2	2	38
SM 42:1;2	5	5	3	13	1	3	30
TAG 50:1	8	3	6	13	6	3	39
SM 34:1;2	26	6	3	12	0	2	49
LPC 18:1	7	5	2	12	4	8	38
LPC 20:4	11	4	1	12	5	7	40
PI 38:3	4	7	3	12	3	3	32
TAG 52:3	7	1	7	11	8	2	36
FFA 18:0	4	17	14	11	3	1	50
PC 36:2	4	9	1	10	2	1	27
SM 42:2;2-isomer1	7	8	2	10	0	1	28
TAG 54:3	6	2	11	10	8	5	42
TAG 54:4	6	0	8	10	9	7	40
TAG 54:2	3	1	12	10	3	2	31
LPC 22:6	22	8	2	9	2	6	49
PC 38:3	14	7	5	9	8	2	45
SM 42:2;2-isomer2	4	13	1	9	4	2	33
PC 34:2	5	8	1	8	3	1	26
SM 42:3;2	5	8	2	7	0	1	23
LPC 20:3-isomer2	25	3	0	6	2	23	59
FFA 20:4	4	25	2	5	1	4	41
SM 36:1;2	13	0	8	4	0	1	26

Table S3. The statistical results of 15 ratios selected by both dynamic concentration and topological structure analyses

Lipids 1	Lipids 2	*p* value					
		C16 vs. M16	C18 vs. M18	C20 vs. M20	M14 vs. M16	M14 vs. M18	M14 vs. M20
LPC 22:6	FFA 20:5	7.52E-04	3.60E-06	2.83E-01	7.60E-03	4.51E-04	1.05E-02
LPC 18:1	FFA 20:5	6.39E-04	3.68E-04	1.52E-02	9.05E-03	4.09E-03	1.29E-02
LPC 20:4	FFA 20:5	2.55E-02	1.22E-03	7.70E-01	6.29E-03	2.35E-03	1.49E-02
LPC 16:0	FFA 20:5	7.25E-03	6.91E-04	4.57E-02	1.59E-02	3.01E-03	7.84E-03
PC 34:2	FFA 20:5	1.45E-03	7.43E-04	4.28E-03	4.17E-02	1.13E-02	2.43E-02
LPC 20:3-isomer2	FFA 20:5	1.81E-02	4.52E-04	4.15E-02	7.96E-03	4.35E-03	1.96E-02
PC 36:3	FFA 20:5	1.12E-03	2.82E-03	2.62E-03	8.32E-02	2.70E-02	5.12E-02
FFA 20:5	TAG 54:3	4.58E-02	1.01E-02	4.27E-01	1.45E-02	4.38E-03	1.64E-02
FFA 20:5	TAG 52:2	1.46E-01	5.33E-02	5.20E-01	7.13E-03	2.00E-03	6.72E-03
FFA 20:5	TAG 52:3	4.65E-01	5.32E-01	4.68E-02	7.75E-03	1.49E-03	4.76E-03
FFA 20:5	TAG 54:4	7.22E-02	2.21E-02	4.60E-01	1.38E-02	3.81E-03	1.44E-02
FFA 20:5	TAG 50:1	2.06E-01	5.04E-02	8.99E-01	7.60E-03	2.00E-03	5.35E-03
FFA 20:5	TAG 54:7-isomer2	1.44E-01	3.79E-02	3.91E-02	6.71E-02	2.51E-03	1.10E-02
FFA 20:5	TAG 54:2	4.25E-02	1.27E-02	7.29E-01	1.23E-02	4.03E-03	1.69E-02
FFA 20:5	TAG 56:7	8.89E-01	9.85E-01	2.18E-01	1.76E-02	1.26E-03	5.23E-03

Reference

1 Hoene, M. *et al.* The lipid profile of brown adipose tissue is sex-specific in mice. *Biochim. Biophys. Acta Mol. Cell Biol. Lipids* **1841**, 1563-1570, (2014).
